# Supplementary material for: A clinically translatable hyperspectral endoscopy (HySE) system for imaging the gastrointestinal tract
Source: Nat Commun. 2019 Apr 23;10:1902. doi: 10.1038/s41467-019-09484-4 (PMC6478902; doi:10.1038/s41467-019-09484-4)
Supplement: Supplementary file 1 — Supplementary Information [file 41467_2019_9484_MOESM1_ESM.pdf]

Supplementary Information for

A clinically translatable hyperspectral endoscopy (HySE) system for imaging the gastrointestinal tract

Yoon et al.

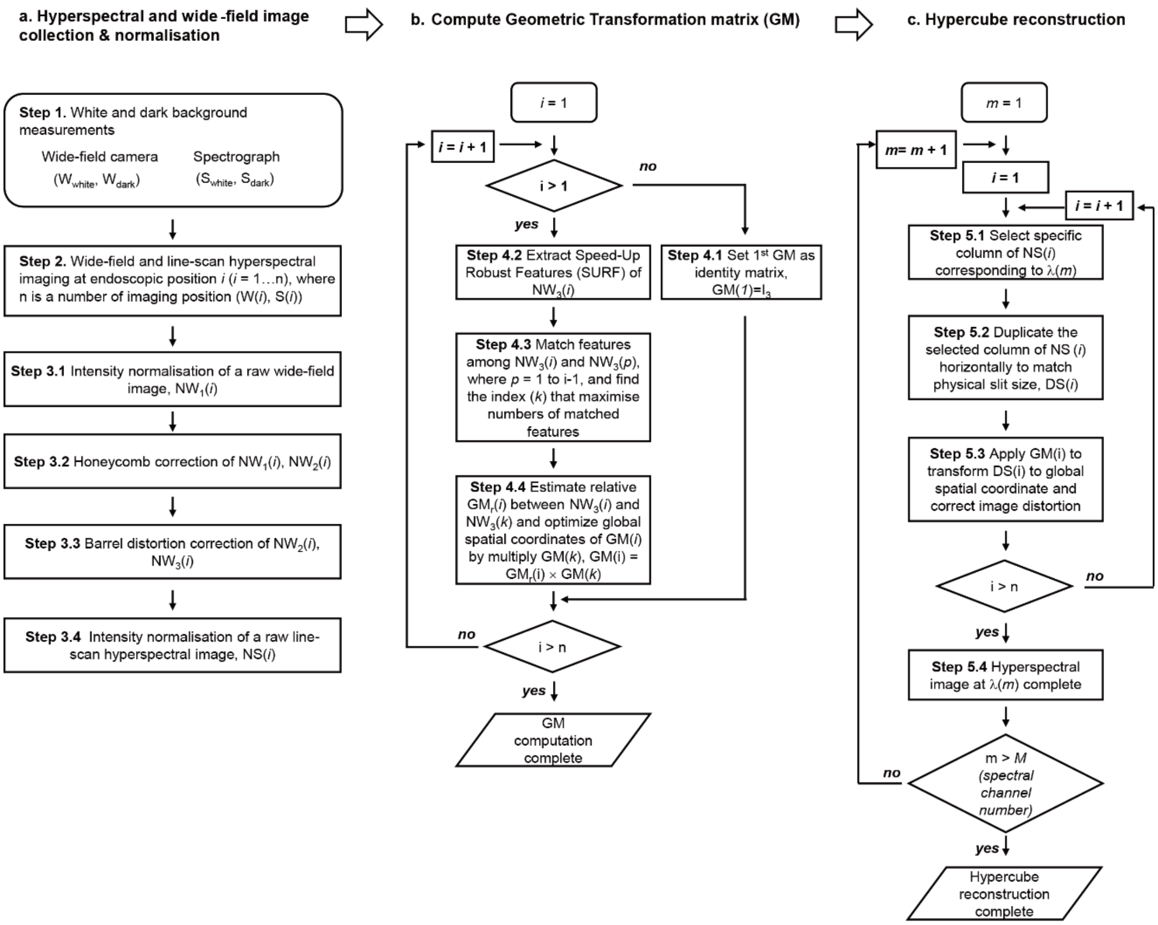

Supplementary Figure. 1. A schematic overview of the workflow of hyperspectral endoscopic imaging. **a** Hyperspectral and wide-field image measurement and normalisation. **b** Wide-field image registration process for estimating geometric transformation matrices (GMs) of each wide-field image to retrieve information about imaging position and distortion required for hypercube reconstruction. **c** Hypercube reconstruction process by exploiting normalised spectral images and estimated GMs.

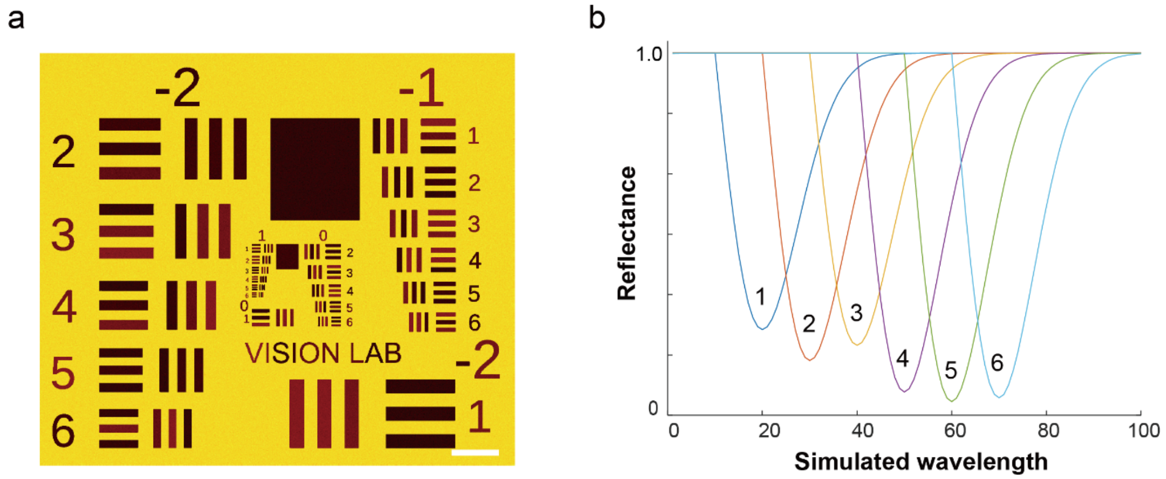

Supplementary Figure. 2. *In silico* USAF 1951 test target used for simulation. a 2D projection image of simulated hyperspectral USAF target along the wavelength. Scale bar = 200 pixels. **b** Six spectral features randomly assigned to each USAF target element.

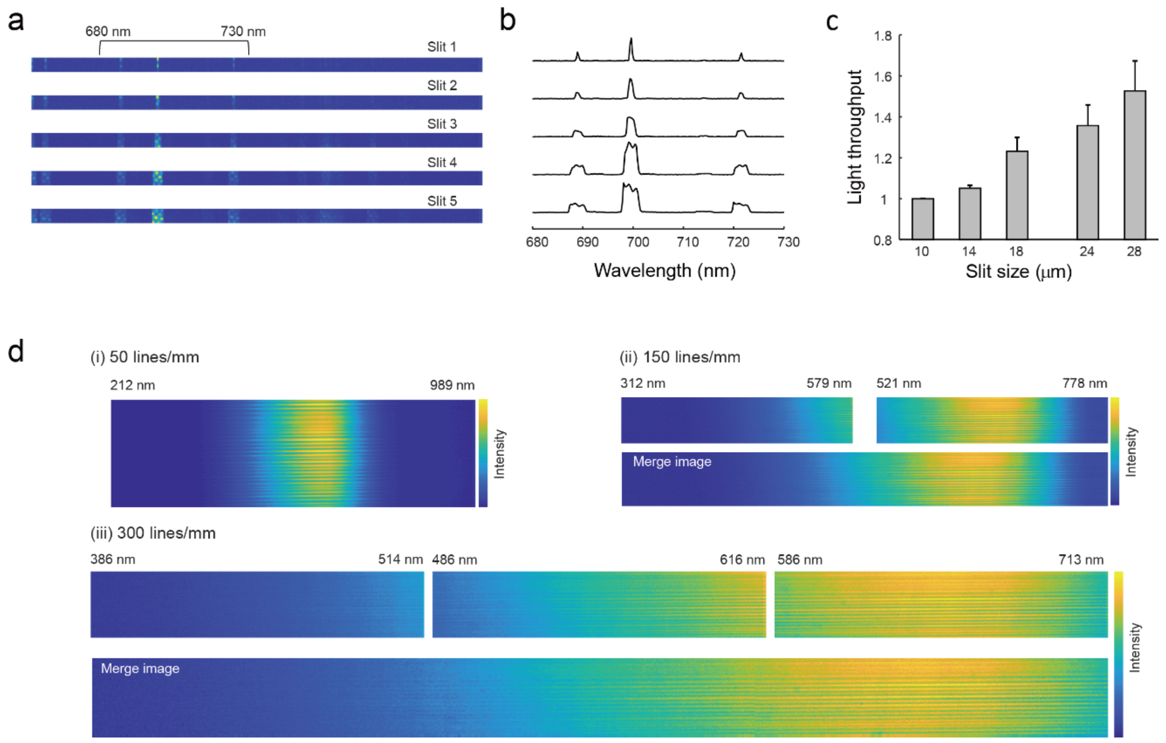

Supplementary Figure. 3. Investigation of spectral resolution and bandwidth. a Spectral images measured using five different slit sizes. **b** Line profiles of spectral images between spectral ranges of 680 to 830 nm. **c** Quantification of light throughput measured using five different slit sizes. Average light throughput measured using the 10 μm slit was used as a reference. Error bars represent standard deviation. **d** Spectral bandwidth of a single spectral images measured using three different gratings. To obtain a spectral image over 300 nm, multiple spectral images were merged.

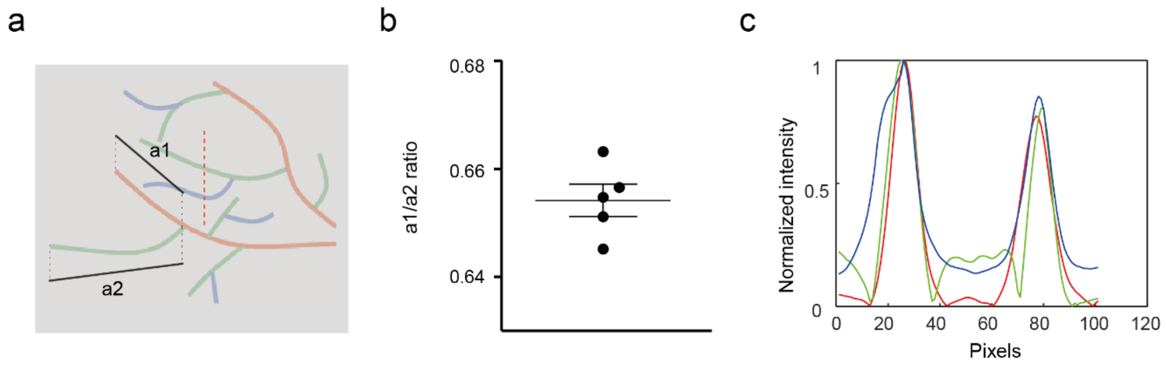

Supplementary Figure. 4. Quantitative analysis of freehand imaging quality. a A vascular tree phantom measured by free-hand hyperspectral imaging. **b** a1 and a2 ratio was quantified shown in a. Centre horizontal line indicates mean value and error bars represent standard deviation. **c** Line profile of the dashed red line shown in a.

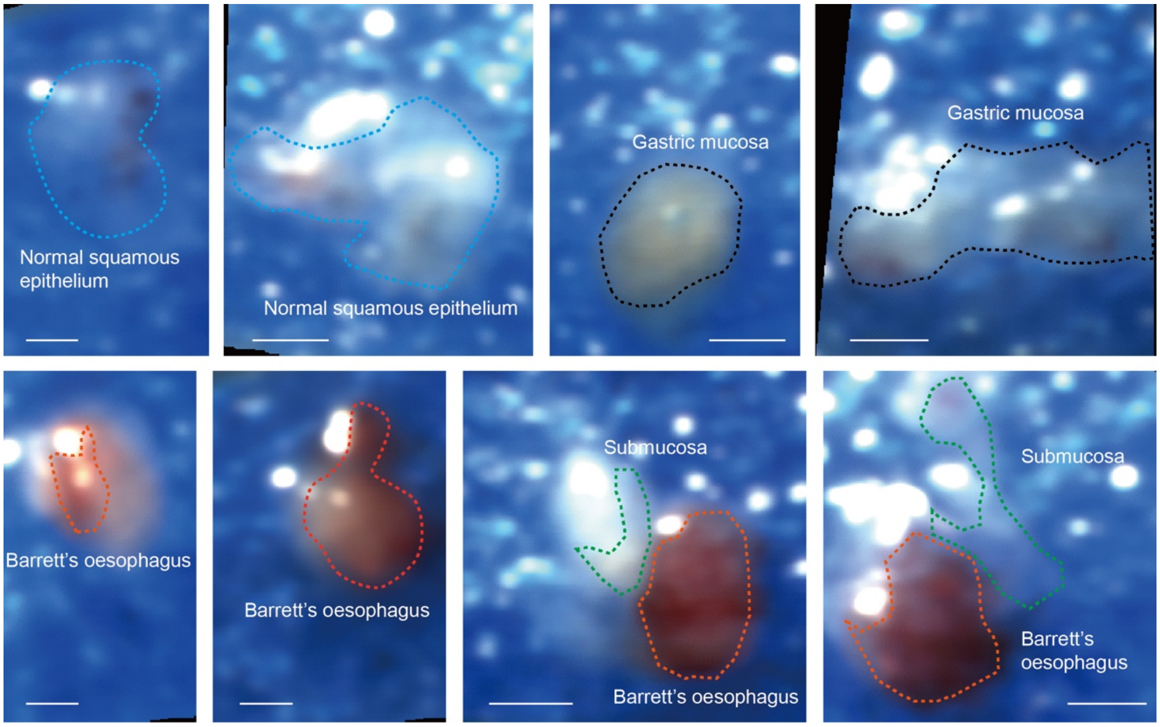

Supplementary Figure. 5. Synthetic RGB images of *ex vivo* human tissues from patients. Dashed line indicates boundary of each tissue type. Scale bars are 1 mm.

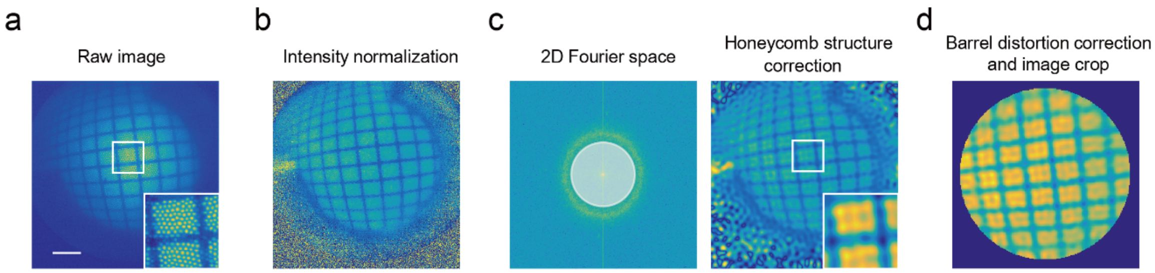

Supplementary Figure. 6. Pre-processing steps for endoscopic image calibration. a Raw endoscopic image. Inset shows magnified image of the white dashed square. Scale bar = 1 mm. **b** Intensity normalized image following correction with white and dark background images. **c** Honeycomb structure correction via low-pass filtering in 2D Fourier space. 2D Fourier transformed image (Left). Grey circle indicates the size of low-pass filtering mask. Image without honeycomb structure obtained by inverse Fourier transformation of filtered 2D Fourier information (Right). Inset shows magnified image of the white dashed square. **d** Image after all pre-processing, including barrel distortion correction and image crop.

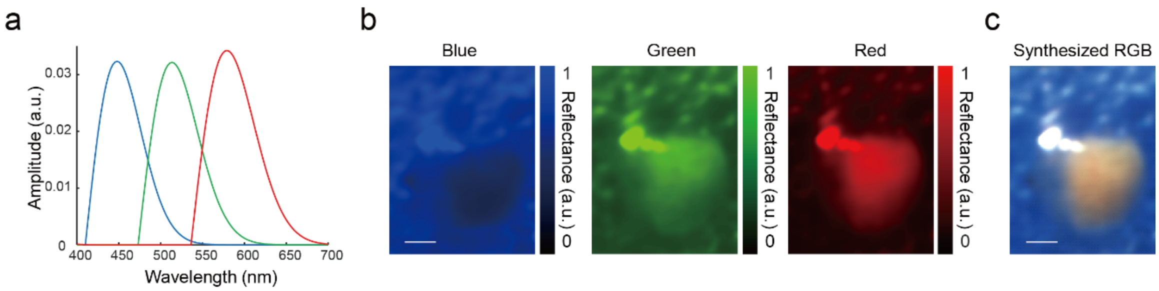

Supplementary Figure. 7. Generation of synthetic RGB image from the hyperspectral image. **a** Spectral profile of applied red, green, and blue filters. **b** Blue, green, and red images of ex vivo human tissue generated by multiplying the corresponding colour filter in a by the reconstructed hypercube. **c** Synthesized RGB image of tissue. Scale bars = 1 mm.

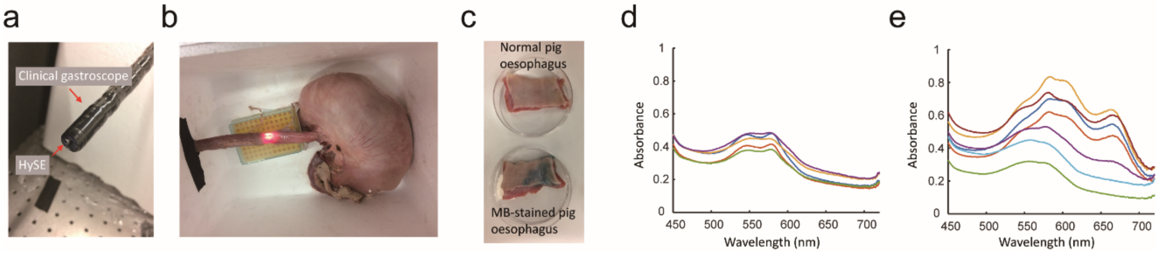

Supplementary Figure. 8. Experiment setup for hyperspectral imaging of an intact *ex vivo* pig oesophagus model. **a** Photography of endoscopy setup. The HySE was inserted via an accessory channel of the clinical gastroscope. **b** Photography of the inflated pig oesophagus and stomach. Illumination at the distal end of the HySE using the internal illumination method can be seen as a bright area in the oesophagus. **c** Photography of the dissected pig oesophagus tissue with and without methylene-blue (MB) staining. Lumen of the pig oesophagus was exposed and measured using a spectrometer. **d, e** Ground-truth absorbance of normal and MB-stained pig oesophagus tissue was measured via the spectrometer at multiple random locations in the tissue.

Supplementary Table 1. Summary of specifications of HySE compared to nearest previous reports.

	HySE			Previous works	
	50 lines/mm	150 lines/mm	300 lines/mm	Ref 15	Ref 54
Operation mode	Spatial scanning			Rotating multicolour filter	Snapshot
Spectral resolution	~ 2.85 ± 0.39 nm	~ 1.10 ± 0.01 nm	~ 0.46 ± 0.05 nm	~ 15 nm	4 to 10 nm
Bandwidth or spectral ranges	750 nm	250 nm	125 nm	400 – 760 nm	450 – 650 nm
Spectral channels	Over 200 channels			18	48
Spatial resolution	119.46 ± 11.58 µm @ working distance of 5 mm			N.A	100 µm
Image acquisition speed	Without pixel binning: 20 fps (spectral imaging)			15 fps	8 to 10 fps
	2 vertical pixels binning: 29 fps (spectral imaging)				
	Over 35 fps (wide-field imaging)				
