# Supplementary material for: A clinically translatable hyperspectral endoscopy (HySE) system for imaging the gastrointestinal tract
Source: Nat Commun. 2019 Apr 23;10:1902. doi: 10.1038/s41467-019-09484-4 (PMC6478902; doi:10.1038/s41467-019-09484-4)
Supplement: Supplementary file 3 — Description of Additional Supplementary Files [file 41467_2019_9484_MOESM3_ESM.pdf]

Description of Additional Supplementary Information

File Name: Supplementary Movie 1

Description: Wide-field image registration process in simulation based on estimation of geometric transformation matrices. Left panel shows an emulated wide-field image with image distortion. Middle panel shows a transformed wide-field image to correct image distortion based on estimated geometric transformation matrices. Right panel shows a registered panoramic image of wide-field images. (Related to Figure 3)

File Name: Supplementary Movie 2

Description: Wide-field image registration and hypercube reconstruction process in freehand HySE operation. Top three images show the printed vascular target, a preprocessed wide-field endoscopic image, and a registered image, respectively. Bottom three images show hypercube reconstruction process at three different wavelengths (495.4, 557.1, and 705.3 nm). (Related to Figure 4)

File Name: Supplementary Movie 3

Description: Wide-field image registration and hypercube reconstruction process of the pig oesophagus model. Top three images show a raw wide-field endoscopic image with the entrance slit position (white square), a registered wide-field image, and a spectral image measured using a 50 lines grating at the centre line of a wide-field image, respectively. Bottom three images show hypercube reconstruction process at three different wavelengths (470.7, 549.3, and 627.8 nm). (Related to Figure 8)
