# Supplementary material for: A clinically translatable hyperspectral endoscopy (HySE) system for imaging the gastrointestinal tract
Source: Nat Commun. 2019 Apr 23;10:1902. doi: 10.1038/s41467-019-09484-4 (PMC6478902; doi:10.1038/s41467-019-09484-4)
Supplement: Supplementary file 7 — Reporting Summary Checklist [file 41467_2019_9484_MOESM7_ESM.pdf]

nature research

Corresponding author(s): *Double-blind peer review submissions: write DBPR and your manuscript number here instead of author names.*

Last updated by author(s): Mar 5, 2019

Reporting Summary

Nature Research wishes to improve the reproducibility of the work that we publish. This form provides structure for consistency and transparency in reporting. For further information on Nature Research policies, see Authors & Referees and the Editorial Policy Checklist.

Statistics

For all statistical analyses, confirm that the following items are present in the figure legend, table legend, main text, or Methods section.

n/a	Confirmed	
☐	☒	The exact sample size (*n*) for each experimental group/condition, given as a discrete number and unit of measurement
☐	☒	A statement on whether measurements were taken from distinct samples or whether the same sample was measured repeatedly
☐	☒	The statistical test(s) used AND whether they are one- or two-sided<br>*Only common tests should be described solely by name; describe more complex techniques in the Methods section.*
☒	☐	A description of all covariates tested
☒	☐	A description of any assumptions or corrections, such as tests of normality and adjustment for multiple comparisons
☐	☒	A full description of the statistical parameters including central tendency (e.g. means) or other basic estimates (e.g. regression coefficient) AND variation (e.g. standard deviation) or associated estimates of uncertainty (e.g. confidence intervals)
☐	☒	For null hypothesis testing, the test statistic (e.g. *F*, *t*, *r*) with confidence intervals, effect sizes, degrees of freedom and *P* value noted<br>*Give P values as exact values whenever suitable.*
☒	☐	For Bayesian analysis, information on the choice of priors and Markov chain Monte Carlo settings
☒	☐	For hierarchical and complex designs, identification of the appropriate level for tests and full reporting of outcomes
☒	☐	Estimates of effect sizes (e.g. Cohen's *d*, Pearson's *r*), indicating how they were calculated

Our web collection on statistics for biologists contains articles on many of the points above.

Software and code

Policy information about availability of computer code

Data collection	1. Spectral image was measured by using Lightfield v6.7 (Princeton Instrument)<br>2. Optical system was controlled by using Labview 2017 (64 bit)<br>3. The spectrometer was controlled by using a commercial software (AvaSoft 8)
Data analysis	1. All data was analysed by using custom Matlab codes and functions (Matlab R2018b)<br>2. All codes are available in the Apollo - University of Cambridge Repository, https://doi.org/10.17863/CAM.17479

For manuscripts utilizing custom algorithms or software that are central to the research but not yet described in published literature, software must be made available to editors/reviewers. We strongly encourage code deposition in a community repository (e.g. GitHub). See the Nature Research guidelines for submitting code & software for further information.

Data

Policy information about availability of data

All manuscripts must include a data availability statement. This statement should provide the following information, where applicable:

- Accession codes, unique identifiers, or web links for publicly available datasets
- A list of figures that have associated raw data
- A description of any restrictions on data availability

The datasets generated during and/or analysed during the current study are available in the Apollo - University of Cambridge Repository, https://doi.org/10.17863/CAM.17479. All other data including raw data are available from the corresponding author upon reasonable request.

Field-specific reporting

Please select the one below that is the best fit for your research. If you are not sure, read the appropriate sections before making your selection.

☒ Life sciences ☐ Behavioural & social sciences ☐ Ecological, evolutionary & environmental sciences

For a reference copy of the document with all sections, see nature.com/documents/nr-reporting-summary-flat.pdf

Life sciences study design

All studies must disclose on these points even when the disclosure is negative.

Sample size	Ex vivo human tissue: 12 tissue samples from 3 patients.
Data exclusions	No data exclusions.
Replication	Ex vivo human tissue was measured in three different days and the spectral profile of all tissue sample showed consistent results, which indicates our experiment is reproducible.
Randomization	Clinicians collected the ex vivo human tissue and gave us without prior information. After completing data analysis, clinicians provided information of tissue based on pathological analysis.
Blinding	The investigators were blinded due to unknown prior information about human tissue.

Reporting for specific materials, systems and methods

We require information from authors about some types of materials, experimental systems and methods used in many studies. Here, indicate whether each material, system or method listed is relevant to your study. If you are not sure if a list item applies to your research, read the appropriate section before selecting a response.

Materials & experimental systems

n/a	Involved in the study	
☒	☐	Antibodies
☒	☐	Eukaryotic cell lines
☒	☐	Palaeontology
☒	☐	Animals and other organisms
☒	☐	Human research participants
☐	☒	Clinical data

Methods

n/a	Involved in the study	
☒	☐	ChIP-seq
☒	☐	Flow cytometry
☒	☐	MRI-based neuroimaging

Clinical data

Policy information about clinical studies

All manuscripts should comply with the ICMJE guidelines for publication of clinical research and a completed CONSORT checklist must be included with all submissions.

Clinical trial registration	The study received ethical approval by the Cambridgeshire 2 Research Ethics Committee (09/H0308/118) and informed consent was obtained from all patients.
Study protocol	*Note where the full trial protocol can be accessed OR if not available, explain why.*
Data collection	The targeted areas of normal (stomach cardia and oesophagus) or diseased mucosa (Barrett's oesophagus or early cancer) were collected using standard endoscopic forceps (Olympus), and endoscopic mucosal resection specimens were collected using a 2 mm diameter punch. All collected samples were positioned with the epithelial layer facing upward in individual containers together with soft sheets of sponge to minimize sample movement during transportation.
Outcomes	*Describe how you pre-defined primary and secondary outcome measures and how you assessed these measures.*
